# Impact of Vascular Endothelial Growth Factor on the Shape, Survival, and Osteogenic Transformation of Gingiva-Derived Stem Cell Spheroids

**DOI:** 10.3390/medicina60122108

**Published:** 2024-12-23

**Authors:** Ji-Eun Lee, Somyeong Hwa, Hee-Ra Lee, Ju-Hwan Kim, Hyun-Jin Lee, Jun-Beom Park

**Affiliations:** 1 Department of Periodontics, College of Medicine, The Catholic University of Korea, Seoul 06591, Republic of Korea; nadi09@naver.com (J.-E.L.); somyeong.hwa@gmail.com (S.H.); yysmjj@naver.com (H.-R.L.); juhwank33@naver.com (J.-H.K.); hyunjinlee0423@gmail.com (H.-J.L.); 2 Department of Periodontics, Korea University Guro Hospital, Seoul 08308, Republic of Korea; 3 Dental Implantology, Graduate School of Clinical Dental Science, The Catholic University of Korea, Seoul 06591, Republic of Korea; 4 Department of Medicine, Graduate School, The Catholic University of Korea, Seoul 06591, Republic of Korea

**Keywords:** cell differentiation, cell survival, osteogenesis, stem cells, vascular endothelial growth factors

## Abstract

*Background and Objectives*: Vascular endothelial growth factor (VEGF) is a protein which stimulates the formation of new blood vessels, playing a crucial role in processes such as wound healing and tumor growth. *Methods*: This study investigated the effects of VEGF on cell viability and osteogenic differentiation in mesenchymal stem cell (MSC) spheroids. Stem cell spheroids were fabricated using concave microwells and cultured with VEGF at concentrations of 0, 0.01, 0.1, 1, and 10 ng/mL. Morphological assessments were conducted on days 1, 3, 5, and 7, while cell viability was evaluated using the LIVE/DEAD assay and Cell Counting Kit-8. Alkaline phosphatase activity (ALP) and calcium deposition were measured to assess osteogenic differentiation, and qPCR was used to analyze osteogenic marker expression. *Results*: The spheroids maintained their shape across all VEGF concentrations, with the largest diameter being at 0.01 ng/mL on day 1, which decreased over time. Cell viability was highest at 0.01 ng/mL VEGF, while calcium deposition peaked at 0.1 ng/mL. Osteogenic markers, including RUNX2, osteocalcin, and COL1A1, showed significant upregulation at 1 ng/mL VEGF. *Conclusions*: These results suggest that VEGF enhances early osteogenic differentiation in MSC spheroids, indicating its potential for bone repair and tissue regeneration. VEGF could be applied in clinical settings for bone healing, fracture repair, and regenerative dentistry treatments.

## 1. Introduction

Bone tissue engineering is an innovative and rapidly advancing field dedicated to the repair and regeneration of damaged bone tissue [1]. This field is built upon a foundation often referred to as the “golden triad”, which consists of three essential components: cells, scaffolds, and growth factors [2]. These elements work in synergy to support the formation of new bone tissue and enhance its structural and functional integration with the surrounding tissues. Growth factors, such as bone morphogenetic proteins, transforming growth factor-β, insulin-like growth factors I and II, platelet-derived growth factor, and both basic and acidic fibroblast growth factors, play a pivotal role in this process by stimulating various cellular activities essential for bone repair and regeneration [3]. Despite significant advancements, bone tissue engineering technology is still evolving, particularly regarding the induction of adequate vascularization within bone defects. Proper vascularization is vital in bone regeneration for several reasons [4]. Blood vessels deliver oxygen, essential nutrients, and regulatory molecules to the regenerating tissue while also removing waste products like carbon dioxide and acids [5]. Furthermore, blood vessels serve as conduits for transporting growth factors which facilitate bone healing and regeneration. Consequently, enhancing vascularization is a primary objective in the development of effective bone regeneration strategies.

Bone tissue engineering shows promise, but several challenges limit its use in clinical settings. It is difficult to mimic the complex structure and strength of natural bone. Many scaffolds lack the right balance of biocompatibility, biodegradability, and mechanical stability [6]. Another key issue is poor vascularization, which affects nutrient supply and waste removal, leading to implant failure. High production costs and strict regulations also make these technologies harder to use widely. Overcoming these challenges will require combining better materials, growth factors, and cell-based approaches.

VEGF is one of the most significant factors in this context due to its crucial role in angiogenesis and vasculogenesis [7]. VEGF is unique because it specifically targets blood vessel cells (endothelial cells), helping them grow, survive, and form new blood vessels. Unlike other growth factors, VEGF is strongly activated by low oxygen levels (hypoxia), making it essential for delivering oxygen to tissues [8]. VEGF is a key player in processes like wound healing and diseases like cancer and eye disorders, where controlling blood vessels is important. VEGF has been shown to stimulate stem cell proliferation, migration, differentiation, and the secretion of other growth factors through the activation of its specific receptors [9]. In addition to its role in angiogenesis, VEGF contributes to osteogenesis, promoting both blood vessel formation and bone tissue development, which are essential for successful bone regeneration [10]. For instance, studies have demonstrated that VEGF enhances both odontogenic and osteogenic differentiation of stem cells in vitro while promoting the formation of mineralized structures in vivo [11]. VEGF signaling also appears to play a role in the osteogenic differentiation of adipose-derived stem cells, although it is not the only pathway involved [4,10]. Additionally, VEGF has been reported to influence the balance between osteoblasts and adipocytes in mesenchymal stem cells within the bone marrow, further underscoring its impact on bone health and regeneration [12].

Stem cells have long been of great interest, especially in the treatment of many diseases [13,14,15]. Stem cell spheroids, assembled as three-dimensional clusters, present substantial potential in tissue engineering, as they more effectively resemble the native three-dimensional organization of tissue compared with standard two-dimensional culture methods [16]. Three-dimensional cultures enable cells to interact in a way that closely resembles their native environment in the body, which can significantly impact their behavior, differentiation potential, and therapeutic efficacy [17]. By providing an environment which enhances cell-to-cell communication, three-dimensional cultures are more effective in simulating tissue development, disease progression, and drug response [18]. Additionally, these cultures have been shown to improve stem cell differentiation into various lineages, including osteogenic, chondrogenic, and neurogenic pathways, highlighting their potential in regenerative medicine applications [16,19].

Given these considerations, it was proposed that adding VEGF could specifically affect the survival and specialization of mesenchymal stem cell spheroids into osteogenic cell types. This research seeks to examine the impact of VEGF on the morphology, survival, osteogenic differentiation, and mineralization of human gingiva-derived mesenchymal stem cells, with the goal of enhancing our understanding of how VEGF can be utilized to optimize bone regeneration strategies in tissue engineering.

## 2. Materials and Methods

### 2.1. Study Design and Cell Source

The purpose of this study was to investigate the effects of VEGF on mesenchymal stem cells (MSCs) derived from gingival tissue. Approval for the research protocol was granted by the Institutional Review Board of Seoul St. Mary’s Hospital in the College of Medicine at the Catholic University of Korea (KC24SISI0503, approved on 26 July 2024, and MC24ZISI0102, approved on 4 October 2024). All experimental procedures adhered to the ethical guidelines outlined in the Declaration of Helsinki. MSCs were isolated from attached keratinized gingival tissues during crown lengthening procedures, and the tissues were de-epithelialized and minced into 1–2 mm^2^ fragments and digested in 0.2 µm filtered alpha-modified minimal essential medium containing dispase and collagenase IV at 37 °C for 30 min. After discarding the first digested cell suspension, the tissues were digested in the same solution for 90 min at 37 °C [20]. The MSCs were cultured in a humidified incubator at 37 °C with an atmosphere of 95% air and 5% CO_2_, with media changes every two to three days to maintain optimal cell growth conditions.

### 2.2. Fabrication of Stem Cell Spheroids

Stem cell spheroids were generated using gingiva-derived MSCs, which were cultured in an osteogenic medium. The cells were seeded onto concave microwells (600 µm in diameter) fabricated from silicon elastomer (StemFIT 3D; MicroFIT, Seongnam-si, Gyeonggi-do, Republic of Korea) at a density of 1 × 10^6^ cells per well [21]. VEGF was added to the osteogenic medium at final concentrations of 0, 0.01, 0.1, 1, and 10 ng/mL (Sigma-Aldrich, St. Louis, MO, USA). The spheroid morphology was monitored and documented on days 1, 3, 5, 7, and 14 using an inverted microscope (CKX41SF, Olympus Corporation, Tokyo, Japan). Spheroid diameters were measured on days 3, 5, and 7 using ImageJ software (Version 1.5, Bethesda, MD, USA) to analyze any morphological changes over time.

### 2.3. Determination of Cell Viability

Cell viability within the spheroids was assessed both qualitatively and quantitatively. Qualitative viability was evaluated on days 1, 3, and 7 using the LIVE/DEAD Kit assay (Molecular Probes, Eugene, OR, USA) [22]. The spheroids were analyzed using a fluorescence microscope (Axiovert 200, Carl Zeiss, Oberkochen, Germany) at a 100× magnification following 60 min of incubation at room temperature. Fluorescence signals are differentiated between living and dead cells based on the selective uptake and binding of specific fluorescent dyes, which exploit differences in cell membrane integrity, metabolic activity, or biochemical composition [23]. In this research, dual staining (LIVE/DEAD assays) was used, which is an often-used combination of dyes such as calcein-AM (for live cells) and propidium iodide (for dead cells), enabling simultaneous detection of both populations in a sample [24]. By using these dyes or combinations in fluorescence microscopy, researchers can reliably differentiate between living and dead cells in various biological assays. Quantitative assessments of cell viability were carried out on days 1, 3, 5, and 7 using Cell Counting Kit-8 (Dojindo, Tokyo, Japan), providing a more detailed assessment of cell viability over time [25].

### 2.4. Assessment of Osteogenic Differentiation

To evaluate osteogenic differentiation, alkaline phosphatase (ALP) activity was analyzed on days 7 and 14 with the aid of an available kit (K412-500, BioVision, Inc., Milpitas, CA, USA) [26]. The cell lysates were incubated with substrate for 30 min at 37 °C, and absorbance was subsequently measured at 405 nm to quantify ALP activity, which serves as an indicator of early osteogenic differentiation. Additionally, calcium deposition, an indicator of bone matrix formation, was assessed through an anthraquinone dye assay [27]. Following a wash and fixation of the spheroids with 70% ethanol, they were stained with Alizarin Red S at room temperature for 30 min, following the manufacturer’s instructions. For quantitative analysis, Alizarin Red was isolated from the samples using cetylpyridinium chloride and analyzed using spectrophotometry at 560 nm. Alizarin Red S was used in the experiments because it specifically stains calcium deposits, making it ideal for studying bone mineralization [28]. It is easy to use, cost-effective, and allows both visualization and quantification of calcium.

### 2.5. Gene Expression Analysis

On day 7, mRNA expression was found using qPCR. Following the manufacturer’s instructions, a commercially available kit (Thermo Fisher Scientific, Inc., Waltham, MA, USA) was used to extract the total RNA. The obtained RNA was then used as a template for reverse transcription, which was carried out with SuperScript II reverse transcriptase (Invitrogen, Carlsbad, CA, USA).

Osteogenic gene expression was assessed on day 7 through a real-time quantitative polymerase chain reaction (qPCR). RNA was isolated from the spheroids using a commercial kit (Thermo Fisher Scientific, Inc., Waltham, MA, USA) according to the manufacturer’s protocol, and its quantity and purity were measured using a spectrophotometer (ND-2000, Thermo Fisher Scientific, Inc.). The integrity of the RNA was further confirmed with a bioanalyzer (Agilent 2100) equipped with an RNA 6000 Nano Chip (Agilent Technologies, Santa Clara, CA, USA). Subsequently, the cDNA was synthesized form the extracted RNA using SuperScript II reverse transcriptase (Invitrogen, Carlsbad, CA, USA).

The specific primers for the target genes, including RUNX2, BSP, OCN, and COL1A1, were designed using sequences obtained from GenBank. The primers used were as follows: RUNX2 (accession no. NM_001015051.3): forward 5′-AAT GAT GGT GTT GAC GCT GA-3’ and reverse 5′-TTG ATA CGT GTG GGA TGT GG-3′, osteocalcin (OCN (accession no. NM_199173.6)), forward 5′-GGTGCAGAGTCCAGCAAAGG-3′ and reverse 5’-GCGCCTGGGTCTCTTCACTA-3′, COL1A1 (accession no. NM_000088.4), forward 5’-CCA GAA GAA CTG GTA CAT CAG CAA-3′ and reverse 5′-CGC CAT ACT CGA ACT GGA ATC-3′, β-actin (housekeeping gene, accession. no. NM 001101), and forward 5′-TGG CAC CCA GCA CAA TGA A-3’ and reverse 5′-CTA AGT CAT AGT CCG CCT AGA AGC A-3′.

### 2.6. Statistical Analysis

The results are presented as the mean ± standard deviation. The assumptions of normality and homogeneity were confirmed, and group differences were evaluated through one-way ANOVA followed by Tukey’s post hoc test, which was applied for subsequent comparisons. Statistical analyses were performed in triplicate for each experimental condition, with significance set at a *p* value <0.05.

## 3. Results

### 3.1. Morphology of Human Gingiva-Derived MSC Spheroids

**Figure 1:**
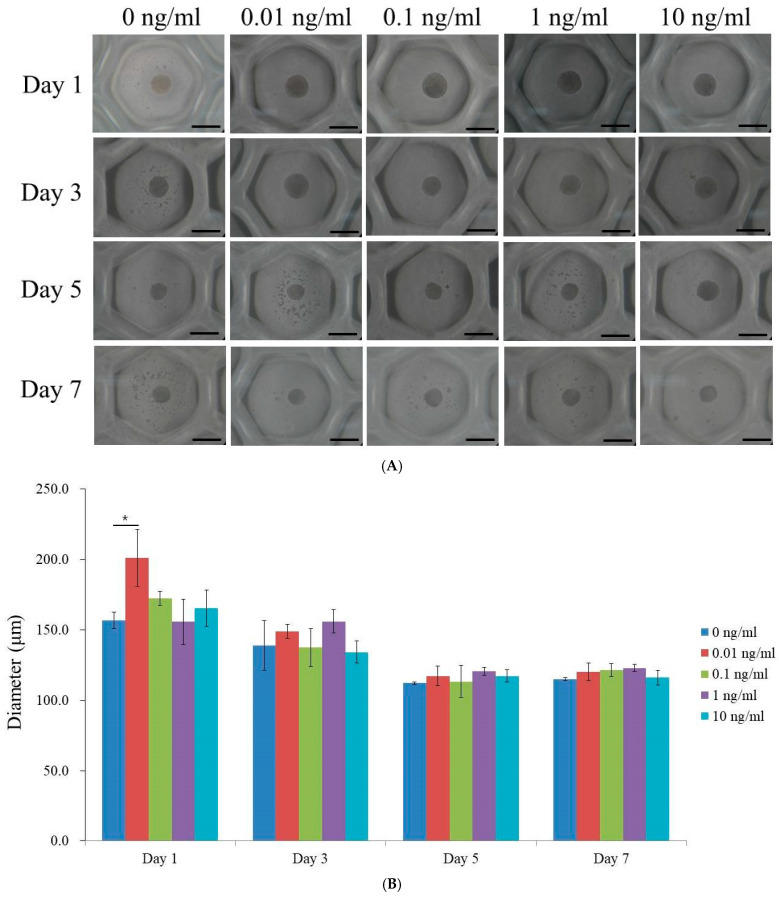
Morphological evaluation. (**A**) The morphologies of the stem cell spheroids treated with VEGF at 0, 0.01, 0.1, 1, and 10 ng/mL on days 1, 3, 5, and 7. The scale bar represents 200 μm (original magnification: ×200). (**B**) The diameters of the stem cell spheroids on days 1, 3, 5, and 7. * *p* < 0.05 on day 1 compared to the time-matched unloaded group.

The morphologies of the human gingiva-derived mesenchymal stem cell spheroids treated with VEGF at concentrations of 0, 0.01, 0.1, 1, and 10 ng/mL on days 1, 3, 5, and 7 are shown in Figure 1A. These images illustrate the shape and structural changes within the spheroids over time, providing a visual representation of how the VEGF concentration influenced the spheroid. Figure 1B quantifies the spheroid diameter from day 1 to day 7, highlighting that the most pronounced difference in spheroid size occurred on day 1, with the 0.01 ng/mL concentration producing the largest spheroids. As the experiment progressed, the differences among all concentrations diminished, with the diameters converging to similar values by day 7. This suggests that the early variations, in response to the VEGF concentration, leveled out over time, leading to consistent spheroid sizes across all treatment groups. Additionally, all VEGF concentrations demonstrated a general reduction in diameter from day 1 to day 7, with no significant outliers.

### 3.2. Assessment of Cell Viability: Qualitative and Quantitative Analysis

**Figure 2:**
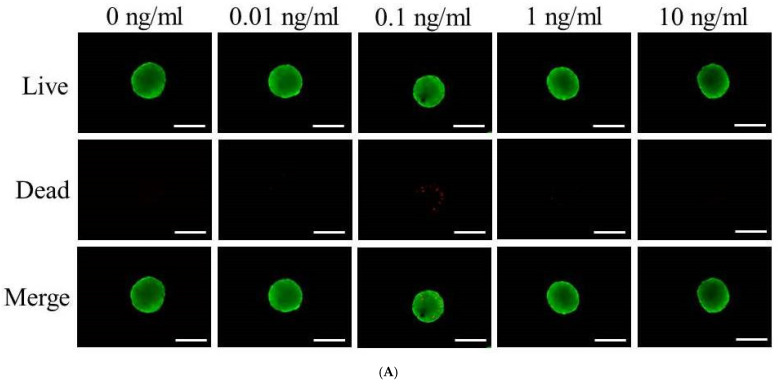

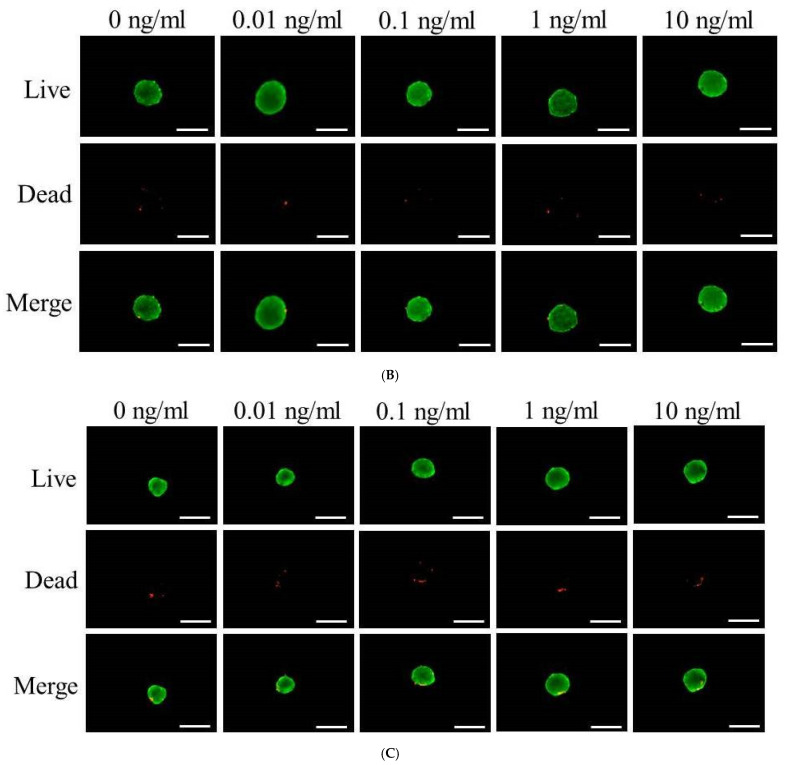

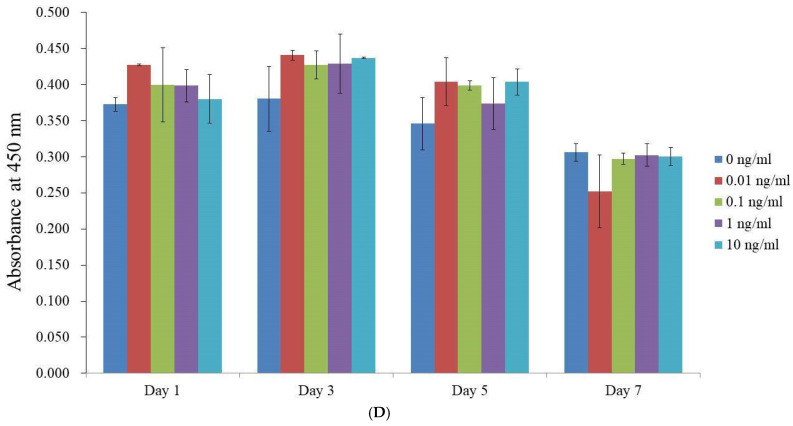
Cellular viability. (**A**) Optical, live, dead, and merged images of stem cell spheroids on day 1. Green represents live cells, while red represents dead cells. Scale bar represents 200 μm (original magnification: ×200). (**B**) Optical, live, dead, and merged images of stem cell spheroids on day 3. Scale bar = 200 μm (original magnification: ×200). (**C**) Optical, live, dead, and merged images of stem cell spheroids on day 7. Scale bar represents 200 μm (original magnification: ×200). (**D**) Cell viability using Cell Counting Kit-8 on days 1, 3, 5, and 7.

Cell viability was qualitatively assessed using a LIVE/DEAD Kit assay on days 1, 3, and 7, with fluorescence microscope images provided in Figure 2A–C. These images displayed cell viability for each VEGF concentration, showcasing the distribution of live and dead cells within the spheroids. Quantitative measurements of cell viability were obtained using the absorbance values (Figure 2D). On day 1, the absorbance values were fairly consistent across all VEGF concentrations, though the 0.01 ng/mL concentration showed slightly higher viability, indicated by its modestly higher absorbance. By day 3, the absorbance values increased for all concentrations, reflecting a general rise in cell viability, as expected during the early culture period. The 0.01 ng/mL concentration continued to demonstrate marginally higher absorbance compared with the other concentrations. By day 7, however, the absorbance values showed a slight decrease relative to day 5, indicating a slight reduction in cell viability as the culture reached a later stage.

### 3.3. Analysis of Alkaline Phosphatase Activity and Calcium Deposition

**Figure 3:**
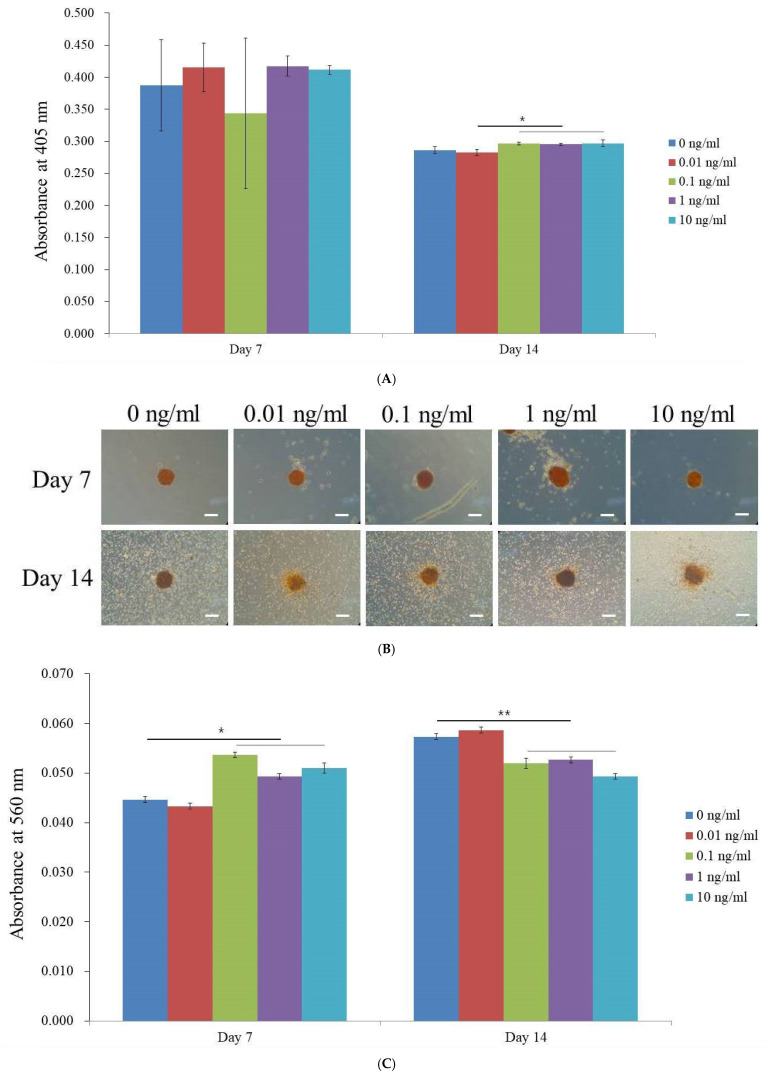
Osteogenic differentiation. (**A**) Alkaline phosphatase activity on days 7 and 14. * *p* < 0.05 on day 14 compared to the time-matched unloaded group. (**B**) Evaluation of calcium deposition in VEGF-treated stem cell spheroids. (**C**) Quantitative analysis of calcium deposition in VEGF-treated stem cell spheroids. * *p* < 0.05 on day 7 compared to the time-matched unloaded group. ** *p* < 0.05 on day 14 compared to the time-matched unloaded group.

On days 7 and 14, the ALP activity was evaluated as an initial marker of osteogenic differentiation (Figure 3A). The data presented in Figure 3B indicate that on day 7, the absorbance values were relatively high for all concentrations, reaching their maximum at 1 ng/mL VEGF, indicating elevated ALP activity at this concentration. At 14 days, the ALP activity exhibited a clear decline across all concentrations, suggesting that osteogenic activity lessened as differentiation proceeded. In addition to ALP activity, calcium deposition was assessed using Alizarin Red S staining, with visible calcium deposits across all treatment groups in Figure 3B. Quantitative data from the anthraquinone dye assay, displayed in Figure 3C, revealed that on day 7, the highest calcium deposition was observed at 0.1 ng/mL VEGF. By day 14, the calcium deposition showed a statistically significant increase at the 0.01 ng/mL concentration (indicated by asterisks (**)), indicating a temporal shift in the optimal VEGF concentration for calcium deposition.

### 3.4. Evaluation of Osteogenic Gene Expression (RUNX2, OCN, and COL1A1) via qPCR

**Figure 4:**
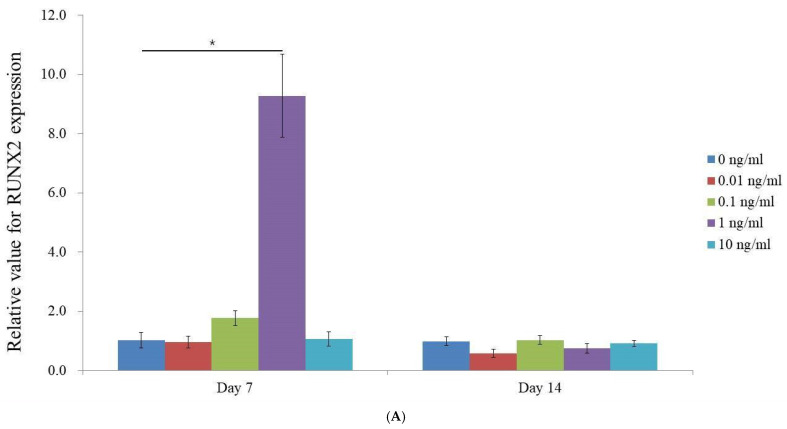

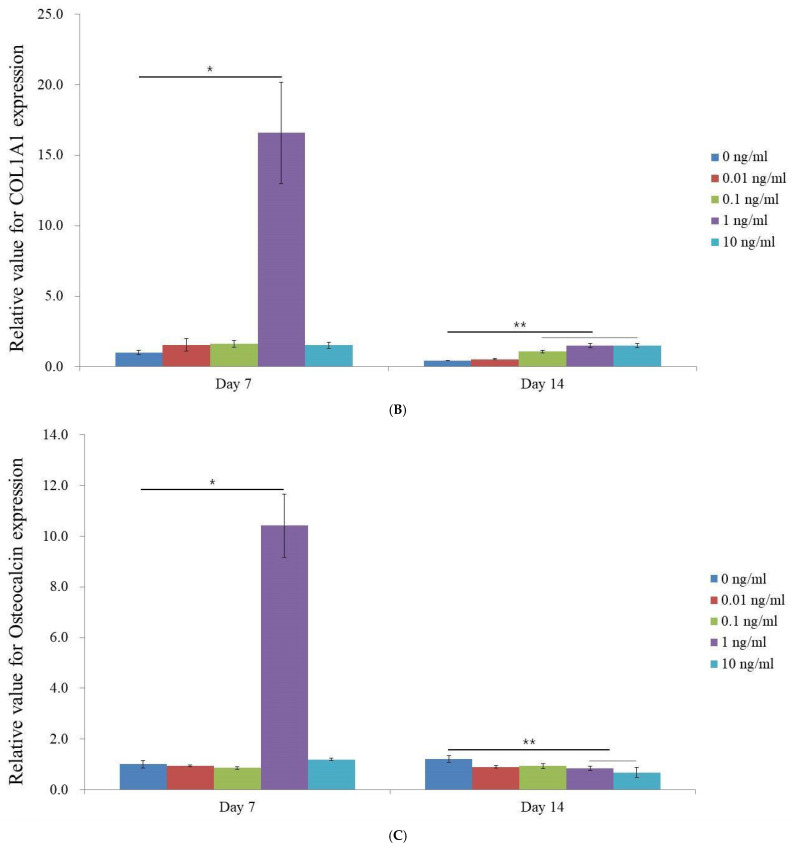
mRNA expression. (**A**) Quantification of expression of RUNX2 mRNA via real-time polymerase chain reaction on days 7 and 14. * *p* < 0.05 on day 7 compared to the time-matched unloaded group. (**B**) Quantification of expression of COL1A1 mRNA via real-time polymerase chain reaction on days 7 and 14. * *p* < 0.05 on day 7 compared to the time-matched unloaded group. ** *p* < 0.05 on day 14 compared to the time-matched unloaded group. (**C**) Quantification of expression of OCN mRNA via real-time polymerase chain reaction on days 7 and 14. * *p* < 0.05 on day 7 compared to the time-matched unloaded group. ** *p* < 0.05 on day 14 compared to the time-matched unloaded group.

The expression levels of osteogenic markers RUNX2, OCN, and COL1A1 were analyzed on day 7 using qPCR (Figure 4A–C). For RUNX2, a critical transcription factor for osteoblast differentiation, the recorded expression levels were as follows: 1.025 ± 0.264, 0.954 ± 0.196, 1.767 ± 0.254, 9.278 ± 1.403, and 1.057 ± 0.248 for VEGF concentrations of 0, 0.01, 0.1, 1, and 10 ng/mL, respectively (Figure 4A). Notably, the 1 ng/mL concentration demonstrated a statistically significant increase in RUNX2 expression on day 7 (*p* < 0.05), while by day 14, the RUNX2 expression levels appeared to be similar across all VEGF concentrations, with no marked differences from the control.

Regarding COL1A1, a key protein in bone matrix formation, its expression levels were reported to be 1.008 ± 0.152, 1.551 ± 0.454, 1.621 ± 0.228, 16.596 ± 3.596, and 1.518 ± 0.214 for VEGF concentrations of 0, 0.01, 0.1, 1, and 10 ng/mL, respectively (Figure 4B). On day 7, the expression of COL1A1 was significantly elevated at 1 ng/mL, with other concentrations showing notably lower levels. By day 14, the expression levels for COL1A1 were generally reduced compared with day 7, although the concentrations of 0.1 ng/mL, 1 ng/mL, and 10 ng/mL showed a moderate increase, with statistically significant differences among these groups.

For OCN, another marker of bone maturation, its expression levels were 1.007 ± 0.153, 0.940 ± 0.036, 0.862 ± 0.046, 10.422 ± 1.241, and 1.191 ± 0.047 for VEGF concentrations of 0, 0.01, 0.1, 1, and 10 ng/mL, respectively (Figure 4C). OCN expression peaked dramatically at 1 ng/mL VEGF on day l, while other concentrations yielded significantly lower levels, underscoring the unique effect of the 1 ng/mL concentration in promoting OCN expression.

## 4. Discussion

In this study, the effects of VEGF on osteogenic differentiation and mineralization were investigated in human mesenchymal stem cell spheroids. The findings show that VEGF is crucial for initiating osteogenesis but has limited long-term effects on cell spheroids. Our data indicate that VEGF boosts early osteogenic markers but loses its effect over time, highlighting the need for additional factors to sustain differentiation.

Human gingiva-derived MSCs are especially advantageous for regenerative medicine applications due to their accessibility, robust proliferation rate, and multipotent differentiation capabilities [29]. These cells also exhibit immunomodulatory and anti-inflammatory properties as well as low risk of tumor formation and promote angiogenesis, making them particularly valuable for therapeutic use. Previous studies have already documented the osteogenic potential of human gingiva-derived MSC spheroids, showing their promise in bone regeneration applications [30].

RUNX2, BSP, OCN, and COL1A1 play key roles in regulating different stages of osteogenesis, the process of bone formation. RUNX2 is a master regulator of osteoblast differentiation which controls the expression of multiple bone-specific genes and is essential for the early stages of osteogenesis [31]. It activates genes like BSP, OCN, and COL1A1, driving the maturation of mesenchymal stem cells into osteoblasts. BSP is a key extracellular matrix protein which promotes mineralization and bone matrix formation. It facilitates calcium and hydroxyapatite binding, which are critical for bone tissue mineralization [32]. OCN is a late-stage marker of osteoblast differentiation and bone formation. OCN regulates bone mineralization and remodeling and acts as a hormone influencing energy metabolism and insulin regulation [33]. COL1A1 encodes the major structural protein of the bone matrix: type I collagen. It provides a scaffold for mineral deposition and structural support during bone formation [34].

Our findings demonstrate that VEGF, particularly at a concentration of 1 ng/mL, effectively promotes early osteogenic differentiation by enhancing the expression of key markers such as RUNX2 and osteocalcin. This supports the notion that rapid vascularization, achievable with low VEGF doses, is critical for improving progenitor survival and proliferation, while higher VEGF doses which increase the vascular density without facilitating early invasion fail to adequately support the metabolic needs of developing tissues [35]. Thus, targeting rapid vascular ingrowth with an optimal low VEGF concentration, such as 1 ng/mL, is more effective for promoting net bone formation than attempting to increase the vascular density with higher VEGF doses. This aligns with existing research demonstrating VEGF’s dual role in osteogenesis and angiogenesis, which are essential for early bone formation. RUNX2, a key factor in osteoblast differentiation, increased with VEGF treatment, suggesting VEGF triggers early osteogenesis in mesenchymal stem cell spheroids. Notably, RUNX2 is part of a genetic program which regulates VEGF expression during endochondral bone formation [36]. However, by day 14, the expression of RUNX2 and other markers such as osteocalcin began to decline, suggesting that VEGF’s influence on osteogenic activity may be temporary. This suggests that VEGF alone cannot sustain osteogenic differentiation and that other signaling pathways are needed in the later stages of bone formation. This finding aligns with the role of RUNX2 as a critical transcription factor for osteoblast differentiation and bone formation [37]. Although VEGF was effective at inducing RUNX2 expression on day 7 at 1 ng/mL, this effect did not persist through day 14, implying that VEGF’s impact may be most crucial during the initial stages of osteogenesis, with other factors becoming more relevant as differentiation advances. COL1A1, a primary component of the extracellular matrix in bone tissue, is associated with matrix production [38], while osteocalcin, a marker of mature osteoblasts, reflects the mineralization and maturation stages of bone development [39]. Previous studies have shown that VEGF mRNA has a relatively short half-life, being approximately 2.44 h in human cervical carcinoma cells, which may explain the transient nature of its effects [40].

The rise in absorbance from day 1 to day 3, followed by stable levels on day 5, indicates effective cell proliferation during this period. The slight decline on day 7 may indicate that cell growth was stabilizing, possibly due to limitations in nutrients or the diminishing effects of VEGF. By day 7, differences in cell viability across the VEGF concentrations were minimal, indicating that VEGF had little impact on cell proliferation in the later stages. The reduction in spheroid diameter over time also reflects changes in cellular viability and cell-to-cell interactions as the culture progressed [41].

Notably, the ALP activity was highest at 1 ng/mL and 0.1 ng/mL on day 7, suggesting that these concentrations are optimal for promoting alkaline phosphatase activity in the early stages of differentiation. By day 14, ALP activity at 0.1 ng/mL, 1 ng/mL, and 10 ng/mL still had an impact on osteogenic differentiation. This pattern, with high ALP activity in the early stages followed by a reduction, is typical of early osteogenic differentiation, indicating that the tested substance had a more pronounced effect on enzyme activity at the initial time points. Additionally, Alizarin Red S staining showed that a concentration of 0.1 ng/mL produced the highest absorbance on day 7, suggesting that this concentration may be optimal for mineralization at this stage. Previous studies have also shown that low VEGF concentrations (e.g., 0.1 ng/mL) can significantly enhance mineralization [42]. By day 14, however, the 0.01 ng/mL treatments showed higher mineralization, suggesting that lower VEGF concentrations may be more beneficial over longer periods.

The decrease in ALP activity and osteogenic gene expression on day 14 likely reflects the natural progression of differentiation from early-stage matrix production to late-stage mineralization, combined with regulatory mechanisms which control osteoblast maturation and the environmental factors inherent in the experimental set-up.

The decrease in spheroid diameter and ALP activity over time suggests that VEGF is most effective during early osteogenic differentiation. As spheroids mature, cells undergo compaction and rearrangement, which could account for the decrease in diameter. Studies have shown that spheroids tended to compact as cell proliferation increased, leading to tighter cell-to-cell interactions and changes in diameter [43]. These results reinforce the notion that VEGF functions primarily as an early osteoblast in the advanced stages of matrix production and mineralization. Other growth factors, such as bone morphogenetic proteins, insulin-like growth factor 2, and platelet-derived growth factor-BB, have been shown to support osteogenic differentiation and mineralization in previous studies [44,45,46]. Additional growth factors like BMPs, TGF-β, or fibroblast growth factor may be needed to sustain differentiation as VEGF’s effects diminish.

A limitation of this study is the reliance on VEGF as the sole growth factor without exploring potential synergistic effects with other osteogenic factors. Future research should investigate the combined effects of VEGF and other signaling molecules such as BMPs, TGF-β, and fibroblast growth factor to achieve a more comprehensive understanding of how these factors interact during bone healing. A strength of this study is the use of 3D spheroid models, which better mimic in vivo cell interactions than 2D cultures. Overall, 3D spheroid stem cell models provide a more realistic and efficient system for studying osteogenesis by mimicking natural tissue environments, improving cell differentiation, and enabling better investigation of bone formation processes and therapeutic interventions [47]. Research has shown that 3D spheroid cultures of gingiva-derived MSCs in osteogenic media have higher ALP activity and mineralization levels than 2D cultures [30]. However, further investigation into more complex tissue-engineered constructs incorporating vascular networks or mechanical stimulation may be necessary to fully replicate in vivo conditions.

One limitation of this study is the reliance on a single growth factor (VEGF) without considering the synergistic effects of other osteogenic factors. Future studies should investigate the combined effects of VEGF with other signaling molecules such as bone morphogenetic proteins, transforming growth factor-β, or fibroblast growth factor to provide a more comprehensive view of how these factors interact in the bone healing process. In addition to the reliance on VEGF as the sole growth factor, this study has other limitations. The analysis was limited to specific time points, which may not fully capture the dynamics of VEGF’s effects on osteogenesis over extended periods. Furthermore, while the spheroid model effectively mimics 3D cell–cell interactions, it does not replicate the complex biomechanical forces and multicellular interactions present in the native bone environment. Finally, the absence of vascular components or immune cell interactions in the current model limits its applicability to fully simulating in vivo conditions. Future studies incorporating long-term observations, mechanical stimulation, and co-cultures with other cell types could provide deeper insights into these processes.

VEGF promotes blood vessel growth (angiogenesis) by encouraging endothelial cells to multiply, move, and form new vessels, ensuring nutrients and oxygen reach tissues. It supports bone formation (osteogenesis) by activating bone-building cells (osteoblasts), recruiting bone precursor cells, and linking blood supply with bone growth. VEGF also works with other growth factors to ensure proper bone repair and development [48]. VEGF initiates osteogenesis effectively but needs additional factors to sustain osteoblast maturation and matrix mineralization during the later stages of bone healing.

The findings of this study demonstrate the potential of VEGF-treated spheroid stem cells in various clinical applications. These include bone fracture healing, where VEGF-treated spheroids could accelerate repair in non-union fractures or critically sized bone defects. In tissue engineering, VEGF-primed spheroids can be integrated into 3D scaffolds to create bioengineered bone grafts for orthopedic and reconstructive surgeries. Also, for osteoporosis treatment, localized delivery of VEGF-enhanced cells may promote bone formation and repair microdamage. In craniofacial and dental applications, VEGF-treated spheroids could aid in craniofacial reconstruction and improve bone integration around dental implants.

Future studies should explore the combined use of VEGF with other growth factors, such as BMPs, TGF-β, and fibroblast growth factor, using sustained release systems to prolong VEGF’s effects and incorporating co-culture models with endothelial or immune cells. Investigating the role of biomechanical stimulation, conducting long-term studies, and validating the findings in in vivo models are also suggested. Furthermore, studies on VEGF isoforms and systematic exploration of combinatorial therapies are recommended to refine strategies for bone regeneration. These additions aim to provide a more comprehensive understanding of VEGF’s role and its potential clinical applications.

## 5. Conclusions

The role of VEGF in the early stages of bone formation was demonstrated through its ability to enhance mesenchymal stem cell differentiation and mineralization, particularly at a concentration of 1 ng/mL. VEGF’s effects were found to be most pronounced during the initial phase of osteogenesis, highlighting its potential in initiating bone regeneration. The need for combining VEGF with other growth factors or employing sustained delivery methods to maintain osteogenic processes over time was identified. The potential of VEGF in early-stage bone tissue engineering was emphasized, along with the necessity for further research on its synergistic effects with other factors and advanced delivery strategies.

## Data Availability

The original contributions presented in the study are included in this article, and further inquiries can be directed to the corresponding author.
